# A Dyadic Technology-Based Intervention for Informal Caregivers and Patients With Dementia

**DOI:** 10.1093/geroni/igab046.012

**Published:** 2021-12-17

**Authors:** Sara Czaja, David Loewenstein, Sarah Weingast

**Affiliations:** 1 Weill Cornell Medicine/Center on Aging and Behavioral Research, New York, New York, United States; 2 University of Miami Miller School of Medicine, Miami, Florida, United States; 3 Weill Cornell Medicine, Weill Cornell Medicine, New York, United States

## Abstract

Most intervention programs in the dementia domain have exclusively focused on the caregiver (CG) or the patient (CR), despite evidence of a reciprocal interaction between the dyad. This presentation will describe a randomized controlled trial that is evaluating the feasibility and efficacy of an innovative dyadic intervention (DT) that is delivered through an interactive technology that includes an evidenced-based CG component, an evidenced-based cognitive training component for the CR and a dyadic component. The program is designed to: be synergistic and emphasize issues important to CGs in the earlier stages of caregiving. The sample involves 200 informal CGs and CRs with early-stage dementia. Data will be presented regarding factors influencing the feasibility of implementing a dyadic intervention such as recruitment challenges (e.g., mutual consent and eligibility), and mutual engagement of both the CG and CR. Strategies implemented to maintain the trial during the COVID-19 pandemic will also be discussed.

